# Increased Peripheral Interleukin 10 Relate to White Matter Integrity in Schizophrenia

**DOI:** 10.3389/fnins.2019.00052

**Published:** 2019-02-07

**Authors:** Gui Fu, Wenjing Zhang, Jing Dai, Jieke Liu, Fei Li, Dongsheng Wu, Yuan Xiao, Chandan Shah, John A. Sweeney, Min Wu, Su Lui

**Affiliations:** ^1^Huaxi MR Research Center, Department of Radiology, West China Hospital of Sichuan University, Chengdu, China; ^2^The Fourth People’s Hospital of Chengdu, Sichuan, China; ^3^Department of Psychiatry and Behavioral Neuroscience, University of Cincinnati, Cincinnati, OH, United States

**Keywords:** schizophrenia, interleukin 10, white matter, inflammation, diffusion tensor imaging

## Abstract

**Background:** Schizophrenia is characterized by the disruption of microstructural white matter (WM) integrity, while the pathogenesis remains unclear. Inflammation has been associated with the WM pathology in schizophrenia. Interleukin 10 (IL-10) has been proven to be related to schizophrenia in both animal and human models. The aim of this study was to explore whether peripheral IL-10 was associated with microstructural WM integrity in schizophrenia.

**Methods:** A total of 47 patients with schizophrenia (SZ) and 49 healthy controls (HC) underwent diffusion tensor imaging and venous blood sampling. Tract-based spatial statistics was conducted to explore the differences in fractional anisotropy (FA), radial diffusivity (RD), mean diffusivity (MD), and axial diffusivity (AD) between patients and controls. A quantitative chemiluminescence assay was performed to measure peripheral IL-10 levels. General linear regression analysis using a stepwise method was applied to examine the relationship between peripheral IL-10 and diffusion measures.

**Results:** Compared with the HC, peripheral IL-10 levels were higher and a significant reduction of FA and AD, and increase of RD and MD were observed in SZ (corrected *p* < 0.05). A regression analysis revealed that peripheral IL-10 was negatively correlated with FA in the right posterior thalamic radiation and left inferior fronto-occipital fasciculus, in SZ (β = -0.51, *p* = 0.01; β = -0.47, *p* = 0.02, respectively) but not in HC (β = -0.01, *p* = 0.95; β = -0.003, *p* = 0.98, respectively), and the differences in regression curves were significant (*z* = 2.50, *p* = 0.01; *z* = 2.37, *p* = 0.02, respectively). IL-10 was negatively connected with MD in the right parietal arcuate fasciculus (β = -0.40, *p* = 0.048) and body of the corpus callosum (β = -0.43, *p* = 0.03) in SZ, while not in HC. The magnitude of correlation in the patient and control group was different (*z* = 2.48, *p* = 0.01 and *z* = 2.61, *p* < 0.01, respectively). In addition, IL-10 was positively correlated with RD in the right parietal arcuate fasciculus in patients (β = 0.45, *p* = 0.04) but not in HC (β = 0.26, *p* = 0.94), but the correlation coefficients were not significant (*z* = 0.98, *p* = 0.32).

**Conclusion:** Our findings demonstrated that elevated peripheral IL-10 levels were associated with the disruption of microstructural WM integrity in schizophrenia, supporting the notion that inflammation plays a regulatory role in the pathology of microstructural WM and is associated with schizophrenia.

## Introduction

Schizophrenia is considered a disconnection disorder characterized by disrupted white matter (WM) integrity (Viher et al., 2016; Di Biase et al., 2017; Kelly et al., 2018). Although the pathogenesis of disrupted WM integrity in schizophrenia remains unclear, neuroinflammation mediated by cytokines appears to be an important pathogenic mechanism (Frodl and Amico, 2014; Najjar and Pearlman, 2015). Moreover, evidence from genomic, blood, postmortem and neuroimaging studies indicates that inflammation plays an important role in the pathophysiological process of schizophrenia (Borovcanin et al., 2015; Najjar and Pearlman, 2015; Fond et al., 2016; Khandaker and Dantzer, 2016; Shivakumar et al., 2018; Xiu et al., 2018). For example, the polymorphisms of interleukin (IL) -10, IL-6 and tumor necrosis factor α (TNF-α) are related to a high risk of developing schizophrenia (Shivakumar et al., 2018; Xiu et al., 2018) and their levels in blood are higher in schizophrenia patients compared with healthy subjects (Kunz et al., 2011; Lee et al., 2017). Elevated expressions of IL-6, IL-1β, IL-8, and SERPINA (a serine protease inhibitor) were associated with a higher WM neuron density below the orbitofrontal cortex in schizophrenia (Fung et al., 2014). Besides, a neuroimaging study found that serum IL-6 and C-reactive protein (CRP) were associated with reduced fractional anisotropy (FA) of WM in schizophrenia, though there was no significant difference in IL-6 and CRP levels between schizophrenia patients and the controls (Prasad et al., 2015). The evidence indicates that the dysregulation of cytokines could lead to the pathophysiological changes of WM in schizophrenia.

Among these cytokines, IL-10, a regulatory cytokine, maintains the balance between pro-inflammatory and anti-inflammatory cytokines (Murray, 2006). Growing evidence has demonstrated that IL-10 is associated with schizophrenia. For example, it has been reported that increased IL-10 expression alleviates behavioral abnormalities in a mouse model (Meyer et al., 2008). In addition, a meta-analysis of genomic studies demonstrated that subjects with a single nucleotide polymorphism (SNP, rs1800872) and two haplotypes (A-C-A and G-C-C) of IL-10 are vulnerable to schizophrenia (Gao et al., 2014). Furthermore, a previous study found an elevation of systemic IL-10 in patients with schizophrenia compared with healthy controls (Kunz et al., 2011). Peripheral IL-10 levels were also correlated to the severity of clinical symptoms of schizophrenia (Xiu et al., 2016). Additionally, it was also observed that atypical antipsychotics could upregulate the blood IL-10 levels (Sugino et al., 2009).

Growing evidence suggests that IL-10 might be associated with WM anomalies (Pang et al., 2005; Vidal et al., 2013). A previous study on rhesus macaques found that higher IL-10 levels in serum were positively associated with WM volume in the regions below the inferior parietal sulcus, at the tail of the lateral lunate sulcus and with WM density in the dorsal prefrontal cortex (Willette et al., 2013). Additionally, *in vivo* studies indicated a link between peripheral IL-10 and microstructural WM integrity. The inflammatory score composited of blood IL-10 and other cytokines including TNF-α, IL-23, and IL-1β was negatively associated with lower FA in patients with Alzheimer’s disease (Swardfager et al., 2017). In addition, peripheral IL-10 was negatively associated with FA and positively associated with radial diffusivity (RD) and mean diffusivity (MD) in patients with bipolar disorder, however, there were no controls in this study (Benedetti et al., 2016). To our knowledge, no *in vivo* study has examined the relationship between systemic IL-10 and microstructural WM integrity in schizophrenia.

Thus, the aim of the present study was to explore whether the changes of peripheral IL-10 were related to the disruption of microstructural WM integrity *in vivo* and clinical symptoms, as well as cognitive ratings in schizophrenia.

## Materials and Methods

### Participants

Forty-seven patients with schizophrenia (28 males and 19 females; mean age [ ± *SD*], 31.85 ± 11.10 year, range: 21–52 year; mean education [ ±*SD*], 12.26 ± 2.76 year; mean duration of illness [ ±*SD*], 7.77 ± 6.99 year) were recruited from the Outpatient Center of the Department of Psychiatry, West China Hospital of Sichuan University. All Patients were diagnosed with schizophrenia using the Structured Clinical Interview for DSM-IV. All patients had received outpatient monotherapy with antipsychotic drugs for at least 6 months and reached the stable phase. All patients were medication free for 10–14 days before the study enrollment.

A total of 49 healthy subjects (22 males and 27 females; mean age [ ±*SD*], 34.63 ± 9.28 year, range: 26–50 year; mean education [ ±*SD*],14.49 ± 4.33 year) were recruited from the local area via the distribution of poster advertisements. They were screened using the non-patient version of the Structured Clinical Interview for DSM-IV, to exclude those with a history of psychiatric illness. Furthermore, none of their first-degree relatives had a known history of psychiatric illness. Exclusion criteria for both groups were: being left handed, history of neurological illness, autoimmune diseases (such as systemic lupus erythematosus, rheumatoid arthritis, and others) or acute infectious illness within the 4 weeks prior to the study, thyroid dysfunction (assessed regularly by measuring the serum triiodothyronine, thyroxine, and thyroid-stimulating hormone in the Outpatient Center), administration of non-steroidal anti-inflammatory drugs or antibiotic drugs within the 4 weeks prior to the study, alcohol or illegal drug dependence/abuse and pregnancy. Magnetic resonance imaging (MRI) scanning, blood sampling and clinical assessment were completed on the same day.

The study was approved by the Ethics Committee of the West China Hospital of Sichuan University. All participants provided written informed consent after being informed about the details of the study.

### MRI Protocol

The MR imaging scans were performed on a 3.0 Tesla Siemens Magnetom Skyra system. The diffusion tensor images (DTIs) were acquired and the parameters were: two b0 images and 60 images with *b*-value of 1000 s/mm; echo time (TE) 93.0 ms, repetition time (TR) 6800 ms, field of view (FOV) 230 mm × 230 mm × 150 mm, flip angle 90°, voxel size 1.8 mm × 1.8 mm × 3.0 mm, slices 50 and slice thickness 3.0 mm. All acquired images were inspected for significant scanning artifacts and gross brain abnormalities and none were observed in any participant.

### Image Processing

The original DICOM images were shifted to NIFTI images using MRIcron software^1^ and were then processed using the FDT toolbox of FSL 5.0.6^2^. Head motion and eddy current were corrected and the non-brain tissues were then removed. Diffusion eigenvectors, eigenvalues and FA were calculated. Tract-based spatial statistic (TBSS^3^) was then conducted for voxel-wise statistical analyses (Smith et al., 2006). Briefly, a non-linear registration tool (FNIRT) was used to register the individual FA map into the standard space. All transformed FA maps were combined into a four-dimensional image, averaged to form mean FA images, and finally a mean FA skeleton template was created. The FA threshold was defined at 0.2 to separate WM and non-WM areas, including gray matter, ventricles, and cerebrospinal fluid. Finally, the registered FA images were projected onto the FA skeleton which created a four-dimensional skeletonized image containing all subjects. Similar analyses were performed for axial diffusivity (AD), MD, and RD.

### Peripheral IL-10 Measurement

Venous blood was collected from all subjects and heparin was the anticoagulant. Within 30 min of collection, blood samples were centrifuged at 1500 rpm for 20 min. Then, plasma samples were stored at -80°C until the concentrations were measured. The peripheral IL-10 concentrations were determined using the Q-Plex^TM^ Custom Assay and were measured twice. The mean values were calculated for the following statistical analysis.

### Clinical Measures

The severity of psychotic symptoms was evaluated by experienced psychiatrists using the Positive and Negative Syndrome Scale (PANSS), which includes three sub-scales (positive symptoms, negative symptoms, and general psychopathology). The cognitive test was conducted by trained and skilled research assistants using the Brief Assessment of Cognition in Schizophrenia (BACS). The BACS tests participants in four domains including verbal memory, processing speed, reasoning and problem solving, and working memory (Keefe et al., 2008). A composite score combining data across subtests was the primary outcome for the following analysis (Eng et al., 2013; Wang et al., 2017).

### Statistical Analysis

The statistical analysis for non-imaging data was conducted using SPSS for windows, version 22.0. The continuous variables were compared using a two-sample *t*-test and the categorical variables were analyzed using a chi-square test. The *p*-value was two tailed at a significance level of < 0.05.

To explore the differences of diffusion parameters between schizophrenia patients and healthy controls, voxel-wise statistical analysis was conducted on the skeletonized images using FSL randomize (Winkler et al., 2014) with 5,000 permutations, with age and gender as covariates. Threshold-free cluster enhancements (TFCE) (Smith and Nichols, 2009) was used for the multiple comparison correction, with a significance level at *p* < 0.05. The Johns Hopkins University International Consortium for Brain Mapping (JHU ICBM-DTI-81) WM labels was used to identify the regions showing group differences in diffusion parameters. To explore the relationship between the diffusion parameters (FA, AD, MD, and RD) and peripheral IL-10 and between diffusion parameters and symptom scores, the DTI values for each subject were extracted from the regions with significant differences between these two groups (cluster size > 50 voxels) (see Supplementary Table S1). The cluster size was defined as the voxels with *p* < 0.05 and *t*-value > 3 and was highly conservative compared with previous studies (Dineen et al., 2009; Meng et al., 2018), based on the calculation method suggested by Bullmore et al. (1999).

Linear regression analysis using a stepwise method was performed to examine the relationship between serum IL-10 levels and DTI values (FA, AD, RD, and MD). The difference of correlation coefficients was calculated with the Fisher Z-Transformation test, via the application of the “cocor” package in R (Diedenhofen and Musch, 2015). In addition, the relationship between IL-10/DTI values and clinical variables (including BMI, illness duration, CPZ equivalent, PANSS scores, and BACS score) was investigated using Pearson correlation. False discovery rate (FDR) was applied for multiple comparison correction and statistical significance was defined as less than 0.05.

In the exploratory analysis, to investigate the differences of diffusion parameters between schizophrenia patients and healthy controls, voxel-wise statistical analysis was conducted on the skeletonized images using FSL randomize with 5,000 permutations, with IL-10 as covariate. Threshold-free cluster enhancements (TFCE) was used for multiple comparison correction, with a significance level at *p* < 0.05. The Johns Hopkins University International Consortium for Brain Mapping (JHU ICBM-DTI-81) WM labels was used to identify the regions showing group differences in diffusion parameters. Findings are available in the Supplementary Materials.

## Results

### Demographic and Clinical Data

The results of demographic and clinical variables are listed in Table 1. There were no significant differences in age and gender between the schizophrenia and control groups. There were differences in BMI (schizophrenia: 23.40 ± 3.19 kg/m^2^; controls: 22.09 ± 3.01 kg/m^2^, *p* = 0.04) and education (schizophrenia: 12.26 ± 2.76 year; controls: 14.49 ± 4.33 year, *p* < 0.01) between the two groups.

**Table 1:** Demographic and clinical variables in patients with schizophrenia and healthy controls.

	Patients Mean (*SD*)	Controls Mean (*SD*)	*p*-Value
Age, years [range]	31.85 (11.10) [21–52]	34.63 (9.28) [26–50]	0.19
No. male/female	28/19	22/27	0.15
Handedness	Right	Right	
BMI, kg/m^2^	23.40 (3.19)	22.09 (3.01)	0.04
Education, years	12.26 (2.76)	14.49 (4.33)	<0.01
Illness duration, years	7.77 (6.99)		
Antipsychotic dosage (CPZ equivalent), mg/d	390.45 (174.76)		
**PANSS**			
Total	59.96 (18.23)		
Positive symptoms	9.71 (4.29)		
Negative symptoms	14.88 (6.76)		
General symptoms	24.96 (8.66)		
**BACS**			
Composite score	36.77 (12.87)		
IL-10, pg/ml	10.59 (0.24)	9.27 (0.24)	<0.001


*BMI, body mass index; CPZ, chlorpromazine; PANSS, Positive and Negative Syndrome Scale; BACS, Brief Assessment of Cognitive in Schizophrenia; SD, standard deviation.*

The levels of serum IL-10 were higher in patients than in controls (schizophrenia: 10.59 ± 0.24 pg/ml; controls: 9.27 ± 0.24 pg/ml, *p* < 0.001).

### TBSS Results

**FIGURE 1:**
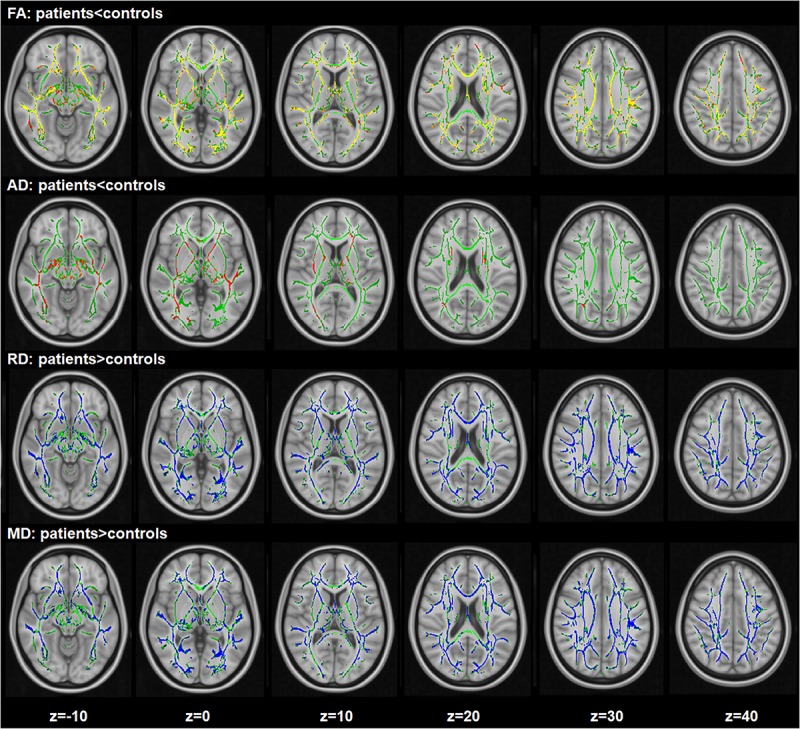
Widespread changes of DTI measures in schizophrenia patients compared with healthy controls. Regions with significant decrease are highlighted in yellow-red/orange. Regions with significant increase are highlighted in blue. Results are shown overlaid on the Montreal Neurologic Institute (MNI) template (1 mm). FA, fractional anisotropy; RD, radial diffusivity; AD, axial diffusivity; MD, mean diffusivity.

Compared with healthy controls, a widespread reduction of FA were observed in patients, including the right superior longitudinal fasciculus, left inferior fronto-occipital fasciculus, bilateral sagittal stratum, corpus callosum (including genu, body, and splenium), bilateral posterior thalamic radiation (including the optic radiation), right posterior corona radiata and the left anterior corona radiata. A global increase in RD was also found in schizophrenia patients in the right sagittal stratum (include inferior longitudinal fasciculus and inferior fronto-occipital fasciculus), left posterior corona radiata, bilateral anterior corona radiata, body and splenium of the corpus callosum, bilateral superior longitudinal fasciculus and the left posterior thalamic radiation (include optic radiation) compared with the healthy controls. A widespread increase of MD was found in schizophrenia patients compared to the controls, with effects in the corpus callosum (including genu, body, and splenium), right superior corona radiata, right anterior corona radiata and the bilateral superior longitudinal fasciculus. In addition, significant AD reduction was found for the clusters in the right superior corona radiata, right anterior corona radiata and body, and the genu of callosum corpus (see Figure 1). In addition, when adjusting for IL-10, the TBSS results of inter-group comparisons were similar with the TBSS findings with age and gender as covariates (see Supplementary Figure S2).

### Regression Analysis Between IL-10 Levels and Diffusion Measures

**FIGURE 2:**
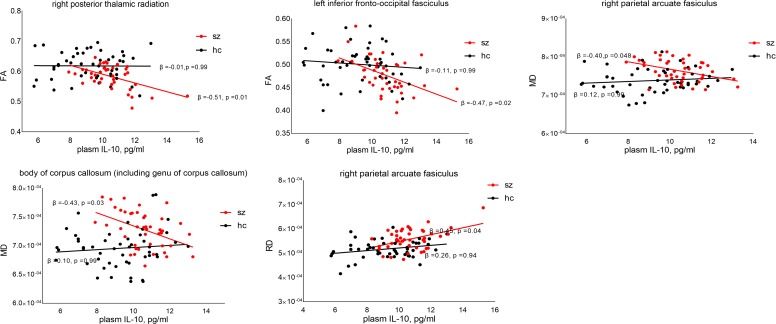
Relationship between peripheral IL-10 and DTI values. *p*-Value was corrected by false discovery rate (FDR). FA, fractional anisotropy; RD, radial diffusivity; MD, mean diffusivity.

Regression analysis showed significant negative correlations between peripheral IL-10 and FA in the right posterior thalamic radiation (β = -0.51, *p* = 0.01) and left inferior fronto-occipital fasciculus (β = -0.47, *p* = 0.02) across patients, but not in the controls (β = -0.01, *p* = 0.95 and β = -0.003, *p* = 0.98, respectively). The correlation coefficients were statistically different between these two groups (*z* = 2.50, *p* = 0.01 and *z* = 2.37, *p* = 0.02, respectively). Significant negative correlations between IL-10 and MD were found in the right parietal arcuate fasciculus (β = -0.39, *p* = 0.048) and the body of the corpus callosum (β = -0.43, *p* = 0.03) in patients, but not in the controls. The correlation coefficients were significantly different (*z* = 2.48, *p* = 0.01 and *z* = 2.61, *p* < 0.01, respectively). In addition, we also found a positive correlation between IL-10 and RD in the right parietal arcuate fasciculus across patients (β = 0.45, *p* = 0.04) but not in the controls (β = 0.26, *p* = 0.94). There was no significant difference in the correlation coefficients (*z* = 0.98, *p* = 0.32) (see Figure 2). No significant correlation was found between AD changes and peripheral IL-10.

### Association Between Demographic/Clinical Factors and Peripheral IL-10/DTI Values

There was no significant correlation between serum IL-10 and clinical variables (including BMI, dosage of antipsychotic medications in CPZ equivalents, PANSS scores, and BACS score). No significant correlation was found between DTI values and changes in illness duration or dosage of antipsychotic medications in CPZ equivalents, PANSS scores, and BACS score (see Supplementary Figure S1).

## Discussion

Focusing on IL-10, a key anti-inflammatory cytokine, the current study provided direct evidence supporting that elevated peripheral IL-10 was related to the disrupted WM integrity of certain bundles in schizophrenia. It therefore provides novel information about the role of cytokines, especially IL-10 in the neuropathology of schizophrenia and adds more evidence relevant to the neuroinflammatory model of schizophrenia.

In our study, systemic IL-10 levels were higher in schizophrenia patients than in healthy subjects, consistent with previous studies (Kunz et al., 2011) and further supporting that the dysregulation of systemic IL-10 is related to the pathogenesis of schizophrenia. Although it remains unclear why the association between systemic IL-10 and WM integrity only existed in schizophrenia patients but not in controls, we assumed that microstructural WM might be more sensitive to IL-10 in patients than healthy subjects. The assumption was supported by the evidence that peripheral cytokines could easily penetrate the blood–brain-barrier (BBB) due to the activated endothelial cell and increased permeability of BBB in schizophrenia (Khandaker and Dantzer, 2016). In addition, a study by Vanessa et al. found that premature newborns with WM injury, who had a high risk of developing mental illness (Baer et al., 2016), had higher IL-10 levels in both cerebrospinal fluid and plasm (Ellison et al., 2005).

How IL-10 might affect microstructural WM integrity remains unclear. Reduced FA values are thought to be related to the disrupted integrity of microstructural WM, impaired axonal membrane and/or demyelination (Beaulieu, 2002). Although the mechanism of reduced FA in schizophrenia is not well-known yet, it is important to note that high IL-10 levels in serum were negatively associated with reduced FA in the posterior thalamic radiata and the inferior fronto-occipital fasciculus in our study. A study *in vitro* found that IL-10 stimulated the activity of macrophage phagocytosis and microglia, which greatly increased myelin phagocytosis (Smith et al., 1998). In addition, a previous study found that IL-10 could enhance initial demyelination via inhibiting the production of interferon γ (IFN-γ) (Puntambekar et al., 2015) while another study suggested that the inflammatory score composited of IL-10, TNF-α, IL-1β, and IL-23 was negatively associated with lower FA within periventricular WM in Alzheimer’s disease (Swardfager et al., 2017). Given that increased RD is related to demyelination (Song et al., 2005), the pattern that higher IL-10 is related to higher RD in schizophrenia, as observed in our study, further supports that IL-10 may be involved in the demyelination of WM. Additionally, IL-10 was also associated with the integrity of the axonal membrane (Atkins et al., 2007). The evidence suggests that WM injury may occur under high serum IL-10 in schizophrenia. However, reduced FA is related not only to myelin and axonal integrity but also to neurofilaments, extracellular water content, and track geometry (Jones et al., 2013). IL-10 has been reported to enhance re-myelination and neuron/axonal growth (Gaupp et al., 2008; Yang et al., 2009), and re-myelination can cause shorter internodes and thinner sheaths, which could increase MD (Peters, 2009; Jelescu et al., 2016). Parallel animal studies are required to clarify the role of IL-10 in these factors. Our study found that by adjusting for IL-10, a widespread FA/AD reduction and MD/RD increase in schizophrenia patients compared with controls. Peripheral IL-10 did not alter the abnormalities of WM in patients but peripheral IL-10 was related to WM integrity, indicating that the peripheral IL-10 may play a regulatory role in the disruption of microstructural WM integrity and the pathogenesis of schizophrenia.

Our study noted that peripheral IL-10 was associated with diffusion measures (FA and MD) in certain bundles including the right posterior thalamic radiation, left inferior fronto-occipital fasciculus, corpus callosum and the arcuate fasciculus. FA reduction of the right posterior thalamic radiation and the left inferior fronto-occipital fasciculus, and MD increase in the corpus callosum have been reported in previous neuroimaging studies of schizophrenia (Fitzsimmons et al., 2013; Gong and He, 2015; Sun et al., 2015; Kelly et al., 2018). The posterior thalamic radiation projects fibers to the parietal, temporal and occipital cortex and processes information on body image (Frieling et al., 2012) while the damage to the posterior thalamic radiation could result in self-perception deficits (Lee et al., 2016). In addition, the inferior fronto-occipital fasciculus connected directly to the frontal and occipital areas (Martino et al., 2010) and reduced FA in this tract was associated with positive symptoms, negative symptoms and auditory-verbal hallucinations in schizophrenia (Curcic-Blake et al., 2015; Oestreich et al., 2016). Furthermore, abnormality of the corpus callosum is associated with reality distortion (i.e., hallucinations and delusions) (Whitford et al., 2010) and negative symptoms in schizophrenia (de Witte et al., 2014). It was also noted that the dysregulation of systemic IL-10 was also related to the negative and general symptoms of schizophrenia (Xiu et al., 2014; Kapelski et al., 2016), as well as cognitive function (Xiu et al., 2016), indicating a link between systemic IL-10, WM integrity and clinical symptoms in schizophrenia. However, our study did not observe a significant association between peripheral IL-10 and clinical assessments in schizophrenia, which might be due to several confounding factors including the relatively small sample size and antipsychotic medication.

The present study had some limitations. First, the sample size was relatively small and studies with larger sample size are required to replicate our findings in the future. Second, the patients in our study were medication free but not drug-naïve, so the potential effects of antipsychotic medication could not be excluded completely. However, once patients were diagnosed with schizophrenia, antipsychotics were immediately administered, before they underwent MRI scanning. Thus, studying patients who are medicated would render findings with a higher generalization. Additionally, investigating pathophysiological changes in patients who are in a clinically stable stage, is also important to identify more convincing findings, since patients at an earlier stage, for example first episode patients, may not have a stable pathophysiology underlying the illness. Third, the cross-sectional nature of our study was limited to determine whether there is a causative relationship between peripheral IL-10 and the alterations of WM integrity in schizophrenia. It is not clear about the longitudinal profile in patients, with respect to the course of schizophrenia. Forth, although IL-10 is a critical cytokine in the immune response, which is a complex process involving many factors. Therefore, future studies investigating more inflammatory mediators and examining their relationship with microstructural WM integrity are necessary for better understanding the pathophysiology of schizophrenia. Fifth, although TBSS analysis is a robust, sensitive and commonly used method in diffusion MR studies, future studies using more methods of DTI analysis are necessary to replicate our findings. Finally, the study only conducted the DTI analysis while an optimized design is necessary to verify the WM abnormalities that could affect DTI results.

In summary, our study revealed that patients with schizophrenia presented elevated peripheral IL-10 levels, which was also related to the deficits of certain WM bundles, including the right posterior thalamic radiation, left inferior fronto-occipital fasciculus, the body of corpus callosum and the arcuate fasciculus. This observation provided *in vivo* evidence supporting the role of IL-10 in structural dysconnectivity, relevant to the neuropathology of schizophrenia.

## Author Contributions

SL, MW, and JS conceived the study and designed the protocol. GF, WZ, JL, JD, DW, and YX conducted the experiments. GF and WZ conducted the statistical analyses. GF, WZ, FL, JS, CS, MW, and SL interpreted the study findings and contributed to developing the manuscript. GF wrote the first draft of the manuscript, which was reviewed by all authors. GF and WZ contributed equally to this work.

## Conflict of Interest Statement

The authors declare that the research was conducted in the absence of any commercial or financial relationships that could be construed as a potential conflict of interest.

## Footnotes

1 http://people.cas.sc.edu/rorden/mricron

2 https://fsl.fmrib.ox.ac.uk/fsl/fslwiki/FSL

3 https://fsl.fmrib.ox.ac.uk/fsl/fslwiki/TBSS

## Supplementary Material

TABLE S1 Extracted regions with significant between-group differences in diffusion measures.

FIGURE S1 Relationship between antipsychotic dosage (CPZ equivalent) and DTI values/ peripheral IL-10 levels. FA, fractional anisotropy; RD, radial diffusivity; MD, mean diffusivity; CPZ, chlorpromazine.

FIGURE S2 Widespread changes of DTI measures in schizophrenia patients compared with healthy controls (peripheral IL-10 as the covariate). Regions with significant decrease are highlighted in yellow-red/orange. Regions with significant increase are highlighted in blue. Results are shown overlaid on the Montreal Neurologic Institute (MNI) template (1 mm). FA, fractional anisotropy; RD, radial diffusivity; AD, axial diffusivity; MD, mean diffusivity.

## References

[B1] Atkins S. Loescher A. R. Boissonade F. M. Smith K. G. Occleston N. O’Kane S. (2007). Interleukin-10 reduces scarring and enhances regeneration at a site of sciatic nerve repair. *J. Peripher. Nerv. Syst.* 12 269–276. 10.1111/j.1529-8027.2007.00148.x 18042137

[B2] Baer R. J. Chambers C. D. Bandoli G. Jelliffe-Pawlowski L. L. (2016). Risk of preterm birth by subtype among Medi-Cal participants with mental illness. *Am. J. Obstet. Gynecol.* 215 519.e1–519.e9. 10.1016/j.ajog.2016.06.017 27329688

[B3] Beaulieu C. (2002). The basis of anisotropic water diffusion in the nervous system – a technical review. *NMR Biomed.* 15 435–455. 10.1002/nbm.782 12489094

[B4] Benedetti F. Poletti S. Hoogenboezem T. A. Mazza E. Ambree O. de Wit H. (2016). Inflammatory cytokines influence measures of white matter integrity in Bipolar Disorder. *J. Affect. Disord.* 202 1–9. 10.1016/j.jad.2016.05.047 27253210

[B5] Borovcanin M. Jovanovic I. Dejanovic S. D. Radosavljevic G. Arsenijevic N. Lukic M. L. (2015). Increase systemic levels of IL-23 as a possible constitutive marker in schizophrenia. *Psychoneuroendocrinology* 56 143–147. 10.1016/j.psyneuen.2015.03.003 25827958

[B6] Bullmore E. T. Suckling J. Overmeyer S. Rabe-Hesketh S. Taylor E. Brammer M. J. (1999). Global, voxel, and cluster tests, by theory and permutation, for a difference between two groups of structural MR images of the brain. *IEEE Trans. Med. Imaging* 18 32–42. 10.1109/42.750253 10193695

[B7] Curcic-Blake B. Nanetti L. van der Meer L. Cerliani L. Renken R. Pijnenborg G. H. (2015). Not on speaking terms: hallucinations and structural network disconnectivity in schizophrenia. *Brain Struct. Funct.* 220 407–418. 10.1007/s00429-013-0663-y 24185461

[B8] de Witte L. Tomasik J. Schwarz E. Guest P. C. Rahmoune H. Kahn R. S. (2014). Cytokine alterations in first-episode schizophrenia patients before and after antipsychotic treatment. *Schizophr. Res.* 154 23–29. 10.1016/j.schres.2014.02.005 24582037

[B9] Di Biase M. A. Cropley V. L. Baune B. T. Olver J. Amminger G. P. Phassouliotis C. (2017). White matter connectivity disruptions in early and chronic schizophrenia. *Psychol. Med.* 47 2797–2810. 10.1017/S0033291717001313 28528586

[B10] Diedenhofen B. Musch J. (2015). cocor: a comprehensive solution for the statistical comparison of correlations. *PLoS One* 10:e0121945. 10.1371/journal.pone.0121945 25835001 PMC4383486

[B11] Dineen R. A. Vilisaar J. Hlinka J. Bradshaw C. M. Morgan P. S. Constantinescu C. S. (2009). Disconnection as a mechanism for cognitive dysfunction in multiple sclerosis. *Brain* 132(Pt 1), 239–249. 10.1093/brain/awn275 18953055

[B12] Ellison V. J. Mocatta T. J. Winterbourn C. C. Darlow B. A. Volpe J. J. Inder T. E. (2005). The relationship of CSF and plasma cytokine levels to cerebral white matter injury in the premature newborn. *Pediatr. Res.* 57 282–286. 10.1203/01.PDR.0000148286.53572.95 15585689

[B13] Eng G. K. Lam M. Bong Y. L. Subramaniam M. Bautista D. Rapisarda A. (2013). Brief assessment of cognition in schizophrenia: normative data in an English-speaking ethnic Chinese sample. *Arch. Clin. Neuropsychol.* 28 845–858. 10.1093/arclin/act060 23912998

[B14] Fitzsimmons J. Kubicki M. Shenton M. E. (2013). Review of functional and anatomical brain connectivity findings in schizophrenia. *Curr. Opin. Psychiatry* 26 172–187. 10.1097/YCO.0b013e32835d9e6a 23324948

[B15] Fond G. Godin O. Brunel L. Aouizerate B. Berna F. Bulzacka E. (2016). Peripheral sub-inflammation is associated with antidepressant consumption in schizophrenia. Results from the multi-center FACE-SZ data set. *J. Affect. Disord.* 191 209–215. 10.1016/j.jad.2015.11.017 26674214

[B16] Frieling H. Fischer J. Wilhelm J. Engelhorn T. Bleich S. Hillemacher T. (2012). Microstructural abnormalities of the posterior thalamic radiation and the mediodorsal thalamic nuclei in females with anorexia nervosa–a voxel based diffusion tensor imaging (DTI) study. *J. Psychiatr. Res.* 46 1237–1242. 10.1016/j.jpsychires.2012.06.005 22770509

[B17] Frodl T. Amico F. (2014). Is there an association between peripheral immune markers and structural/functional neuroimaging findings? *Prog. Neuropsychopharmacol. Biol. Psychiatry* 48 295–303. 10.1016/j.pnpbp.2012.12.013 23313563

[B18] Fung S. J. Joshi D. Fillman S. G. Weickert C. S. (2014). High white matter neuron density with elevated cortical cytokine expression in schizophrenia. *Biol. Psychiatry* 75 e5–e7. 10.1016/j.biopsych.2013.05.031 23830667

[B19] Gao L. Li Z. Chang S. Wang J. (2014). Association of interleukin-10 polymorphisms with schizophrenia: a meta-analysis. *PLoS One* 9:e90407. 10.1371/journal.pone.0090407 24603720 PMC3946087

[B20] Gaupp S. Cannella B. Raine C. S. (2008). Amelioration of experimental autoimmune encephalomyelitis in IL-4Ralpha-/- mice implicates compensatory up-regulation of Th2-type cytokines. *Am. J. Pathol.* 173 119–129. 10.2353/ajpath.2008.071156 18535177 PMC2438290

[B21] Gong Q. He Y. (2015). Depression, neuroimaging and connectomics: a selective overview. *Biol. Psychiatry* 77 223–235. 10.1016/j.biopsych.2014.08.009 25444171

[B22] Jelescu I. O. Zurek M. Winters K. V. Veraart J. Rajaratnam A. Kim N. S. (2016). In vivo quantification of demyelination and recovery using compartment-specific diffusion MRI metrics validated by electron microscopy. *Neuroimage* 132 104–114. 10.1016/j.neuroimage.2016.02.004 26876473 PMC4851889

[B23] Jones D. K. Knosche T. R. Turner R. (2013). White matter integrity, fiber count, and other fallacies: the do’s and don’ts of diffusion MRI. *Neuroimage* 73 239–254. 10.1016/j.neuroimage.2012.06.081 22846632

[B24] Kapelski P. Skibinska M. Maciukiewicz M. Pawlak J. Zaremba D. Twarowska-Hauser J. (2016). Family-based association study of interleukin 10 (IL10) and interleukin 10 receptor alpha (IL10RA) functional polymorphisms in schizophrenia in Polish population. *J. Neuroimmunol.* 297 92–97. 10.1016/j.jneuroim.2016.05.010 27397081

[B25] Keefe R. S. Harvey P. D. Goldberg T. E. Gold J. M. Walker T. M. Kennel C. (2008). Norms and standardization of the Brief Assessment of Cognition in Schizophrenia (BACS). *Schizophr. Res.* 102 108–115. 10.1016/j.schres.2008.03.024 18495435

[B26] Kelly S. Jahanshad N. Zalesky A. Kochunov P. Agartz I. Alloza C. (2018). Widespread white matter microstructural differences in schizophrenia across 4322 individuals: results from the ENIGMA Schizophrenia DTI Working Group. *Mol. Psychiatry* 23 1261–1269. 10.1038/mp.2017.170 29038599 PMC5984078

[B27] Khandaker G. M. Dantzer R. (2016). Is there a role for immune-to-brain communication in schizophrenia? *Psychopharmacology* 233 1559–1573. 10.1007/s00213-015-3975-1 26037944 PMC4671307

[B28] Kunz M. Cereser K. M. Goi P. D. Fries G. R. Teixeira A. L. Fernandes B. S. (2011). Serum levels of IL-6, IL-10 and TNF-alpha in patients with bipolar disorder and schizophrenia: differences in pro- and anti-inflammatory balance. *Braz. J. Psychiatr.* 33 268–274. 10.1590/S1516-44462011000300010 21971780

[B29] Lee E. E. Hong S. Martin A. S. Eyler L. T. Jeste D. V. (2017). Inflammation in schizophrenia: cytokine levels and their relationships to demographic and clinical variables. *Am. J. Geriatr. Psychiatry* 25 50–61. 10.1016/j.jagp.2016.09.009 27840055 PMC5164855

[B30] Lee S. J. Kim B. Oh D. Kim M. K. Kim K. H. Bang S. Y. (2016). White matter alterations associated with suicide in patients with schizophrenia or schizophreniform disorder. *Psychiatry Res. Neuroimaging* 248 23–29. 10.1016/j.pscychresns.2016.01.011 26774424

[B31] Martino J. Brogna C. Robles S. G. Vergani F. Duffau H. (2010). Anatomic dissection of the inferior fronto-occipital fasciculus revisited in the lights of brain stimulation data. *Cortex* 46 691–699. 10.1016/j.cortex.2009.07.015 19775684

[B32] Meng L. Chen Y. Xu X. Chen T. Lui S. Huang X. (2018). The neurobiology of brain recovery from traumatic stress: a longitudinal DTI study. *J. Affect. Disord.* 225 577–584. 10.1016/j.jad.2017.08.075 28886498

[B33] Meyer U. Murray P. J. Urwyler A. Yee B. K. Schedlowski M. Feldon J. (2008). Adult behavioral and pharmacological dysfunctions following disruption of the fetal brain balance between pro-inflammatory and IL-10-mediated anti-inflammatory signaling. *Mol. Psychiatry* 13 208–221. 10.1038/sj.mp.4002042 17579604

[B34] Murray P. J. (2006). Understanding and exploiting the endogenous interleukin-10/STAT3-mediated anti-inflammatory response. *Curr. Opin. Pharmacol.* 6 379–386. 10.1016/j.coph.2006.01.010 16713356

[B35] Najjar S. Pearlman D. M. (2015). Neuroinflammation and white matter pathology in schizophrenia: systematic review. *Schizophr. Res.* 161 102–112. 10.1016/j.schres.2014.04.041 24948485

[B36] Oestreich L. K. McCarthy-Jones S. Whitford T. J. (2016). Decreased integrity of the fronto-temporal fibers of the left inferior occipito-frontal fasciculus associated with auditory verbal hallucinations in schizophrenia. *Brain Imaging Behav.* 10 445–454. 10.1007/s11682-015-9421-5 26112051

[B37] Pang Y. Rodts-Palenik S. Cai Z. Bennett W. A. Rhodes P. G. (2005). Suppression of glial activation is involved in the protection of IL-10 on maternal *E. coli* induced neonatal white matter injury. *Brain Res. Dev. Brain Res.* 157 141–149. 10.1016/j.devbrainres.2005.03.015 15878785

[B38] Peters A. (2009). The effects of normal aging on myelinated nerve fibers in monkey central nervous system. *Front. Neuroanat.* 3:11. 10.3389/neuro.05.011.2009 19636385 PMC2713738

[B39] Prasad K. M. Upton C. H. Nimgaonkar V. L. Keshavan M. S. (2015). Differential susceptibility of white matter tracts to inflammatory mediators in schizophrenia: an integrated DTI study. *Schizophr. Res.* 161 119–125. 10.1016/j.schres.2014.09.043 25449712 PMC4277723

[B40] Puntambekar S. S. Hinton D. R. Yin X. Savarin C. Bergmann C. C. Trapp B. D. (2015). Interleukin-10 is a critical regulator of white matter lesion containment following viral induced demyelination. *Glia* 63 2106–2120. 10.1002/glia.22880 26132901 PMC4755156

[B41] Shivakumar V. Debnath M. Venugopal D. Rajasekaran A. Kalmady S. V. Subbanna M. (2018). Influence of correlation between HLA-G polymorphism and Interleukin-6 (IL6) gene expression on the risk of schizophrenia. *Cytokine* 107 59–64. 10.1016/j.cyto.2017.11.016 29217401

[B42] Smith M. E. van der Maesen K. Somera F. P. (1998). Macrophage and microglial responses to cytokines in vitro: phagocytic activity, proteolytic enzyme release, and free radical production. *J. Neurosci. Res.* 54 68–78. 10.1002/(sici)1097-4547(19981001)54 9778151

[B43] Smith S. M. Jenkinson M. Johansen-Berg H. Rueckert D. Nichols T. E. Mackay C. E. (2006). Tract-based spatial statistics: voxelwise analysis of multi-subject diffusion data. *Neuroimage* 31 1487–1505. 10.1016/j.neuroimage.2006.02.024 16624579

[B44] Smith S. M. Nichols T. E. (2009). Threshold-free cluster enhancement: addressing problems of smoothing, threshold dependence and localisation in cluster inference. *Neuroimage* 44 83–98. 10.1016/j.neuroimage.2008.03.061 18501637

[B45] Song S. K. Yoshino J. Le T. Q. Lin S. J. Sun S. W. Cross A. H. (2005). Demyelination increases radial diffusivity in corpus callosum of mouse brain. *Neuroimage* 26 132–140. 10.1016/j.neuroimage.2005.01.028 15862213

[B46] Sugino H. Futamura T. Mitsumoto Y. Maeda K. Marunaka Y. (2009). Atypical antipsychotics suppress production of proinflammatory cytokines and up-regulate interleukin-10 in lipopolysaccharide-treated mice. *Prog. Neuropsychopharmacol. Biol. Psychiatry* 33 303–307. 10.1016/j.pnpbp.2008.12.006 19138716

[B47] Sun H. Lui S. Yao L. Deng W. Xiao Y. Zhang W. (2015). Two patterns of white matter abnormalities in medication-naive patients with first-episode schizophrenia revealed by diffusion tensor imaging and cluster analysis. *JAMA Psychiatry* 72 678–686. 10.1001/jamapsychiatry.2015.0505 25993492

[B48] Swardfager W. Yu D. Ramirez J. Cogo-Moreira H. Szilagyi G. Holmes M. F. (2017). Peripheral inflammatory markers indicate microstructural damage within periventricular white matter hyperintensities in Alzheimer’s disease: a preliminary report. *Alzheimers Dement.* 7 56–60. 10.1016/j.dadm.2016.12.011 28275700 PMC5328682

[B49] Vidal P. M. Lemmens E. Dooley D. Hendrix S. (2013). The role of “anti-inflammatory” cytokines in axon regeneration. *Cytokine Growth Factor Rev.* 24 1–12. 10.1016/j.cytogfr.2012.08.008 22985997

[B50] Viher P. V. Stegmayer K. Giezendanner S. Federspiel A. Bohlhalter S. Vanbellingen T. (2016). Cerebral white matter structure is associated with DSM-5 schizophrenia symptom dimensions. *Neuroimage Clin.* 12 93–99. 10.1016/j.nicl.2016.06.013 27408794 PMC4925890

[B51] Wang L. J. Huang Y. C. Hung C. F. Chen C. K. Chen Y. C. Lee P. Y. (2017). The Chinese version of the brief assessment of cognition in schizophrenia: data of a large-scale mandarin-speaking population. *Arch. Clin. Neuropsychol.* 32 289–296. 10.1093/arclin/acw100 28431029

[B52] Whitford T. J. Kubicki M. Schneiderman J. S. O’Donnell L. J. King R. Alvarado J. L. (2010). Corpus callosum abnormalities and their association with psychotic symptoms in patients with schizophrenia. *Biol. Psychiatry* 68 70–77. 10.1016/j.biopsych.2010.03.025 20494336 PMC2900500

[B53] Willette A. A. Coe C. L. Birdsill A. C. Bendlin B. B. Colman R. J. Alexander A. L. (2013). Interleukin-8 and interleukin-10, brain volume and microstructure, and the influence of calorie restriction in old rhesus macaques. *Age* 35 2215–2227. 10.1007/s11357-013-9518-y 23463321 PMC3825005

[B54] Winkler A. M. Ridgway G. R. Webster M. A. Smith S. M. Nichols T. E. (2014). Permutation inference for the general linear model. *Neuroimage* 92 381–397. 10.1016/j.neuroimage.2014.01.060 24530839 PMC4010955

[B55] Xiu M. H. Man L. J. Wang D. Du X. Yin G. Zhang Y. (2018). Tumor necrosis factor-alpha -1031T/C polymorphism is associated with cognitive deficits in chronic schizophrenia patients versus healthy controls. *Am. J. Med. Genet. B Neuropsychiatr. Genet.* 177 379–387. 10.1002/ajmg.b.32622 29633506

[B56] Xiu M. H. Tian L. Chen S. Tan Y. L. Chen D. C. Chen J. (2016). Contribution of IL-10 and its -592 A/C polymorphism to cognitive functions in first-episode drug-naive schizophrenia. *Brain Behav. Immun.* 57 116–124. 10.1016/j.bbi.2016.03.005 26971470

[B57] Xiu M. H. Yang G. G. Tan Y. L. Chen D. C. Tan S. P. Wang Z. R. (2014). Decreased interleukin-10 serum levels in first-episode drug-naive schizophrenia: relationship to psychopathology. *Schizophr. Res.* 156 9–14. 10.1016/j.schres.2014.03.024 24766914

[B58] Yang J. Jiang Z. Fitzgerald D. C. Ma C. Yu S. Li H. (2009). Adult neural stem cells expressing IL-10 confer potent immunomodulation and remyelination in experimental autoimmune encephalitis. *J. Clin. Invest.* 119 3678–3691. 10.1172/JCI37914 19884657 PMC2786785

