# Supplementary material for: Increased Peripheral Interleukin 10 Relate to White Matter Integrity in Schizophrenia
Source: Front Neurosci. 2019 Feb 7;13:52. doi: 10.3389/fnins.2019.00052 (PMC6374337; doi:10.3389/fnins.2019.00052)
Supplement: TABLE S1 — Extracted regions with significant between-group differences in diffusion measures. [file Table_1.docx]

**Supplementary Table 1. Extracted regions with significant between-group differences in diffusion measures.**

| Regions | Peak MNI coordinate | | | Voxels | Patients | Controls |
| --- | --- | --- | --- | --- | --- | --- |
|  | x | y | z |  | FA value, mean(*s.d.*) | |
| Anterior corona radiata.L | 112 | 160 | 78 | 611 | 0.486(0.038) | 0.515(0.032) |
| Sagittal striatum (include inferior logitudinal fasciculus and inferior fronto-occipital fasciculus).R | 53 | 75 | 73 | 282 | 0.567(0.024) | 0.595(0.036) |
| Body of corpus callosum | 102 | 135 | 101 | 278 | 0.628(0.050) | 0.659(0.041) |
| Posterior corona radiata.R | 62 | 64 | 100 | 264 | 0.460(0.032) | 0.493(0.034) |
| Splenium of corpus callosum | 110 | 73 | 96 | 211 | 0.669(0.038) | 0.493(0.034) |
| Sagittal striatum (include inferior logitudinal fasciculus and inferior fronto-occipital fasciculus).L | 132 | 108 | 53 | 139 | 0.563(0.034) | 0.598(0.034) |
| Splenium of corpus callosum | 72 | 82 | 106 | 118 | 0.501(0.027) | 0.518(0.024) |
| Body of corpus callosum | 81 | 141 | 95 | 115 | 0.691(0.058) | 0.716(0.041) |
| Temporal arcuate fasciculus.L | 36 | 96 | 62 | 98 | 0.388(0.036) | 0.418(0.035) |
| Posterior thalamic radiation (include optic radiation).L | 123 | 65 | 91 | 89 | 0.493(0.047) | 0.525(0.049) |
| Inferior fronto-occipital fasciculus.L | 111 | 143 | 59 | 73 | 0.477(0.038) | 0.501(0.039) |
| Posterior thalamic radiation (include optic radiation).R | 60 | 64 | 73 | 69 | 0.584(0.038) | 0.618(0.046) |
| Superior longitudinal fasciculus.R | 52 | 119 | 103 | 63 | 0.482(0.029) | 0.512(0.042) |
| Splenium of corpus callosum | 64 | 73 | 83 | 59 | 0.740(0.054) | 0.777(0.037) |
| Body of corpus callosum | 98 | 145 | 92 | 51 | 0.746(0.042) | 0.771(0.037) |
|  |  |  |  |  | AD value, mean(*s.d.*) | |
| Superior corona radiata.R | 63 | 125 | 101 | 207 | 1.136(0.058) | 1.199(0.084) |
| Anterior corona radiata.R | 66 | 146 | 103 | 137 | 0.987(0.047) | 1.033(0.062) |
| Superior corona radiata.R | 113 | 66 | 102 | 129 | 1.220(0.053) | 1.260(0.052) |
| Superior corona radiata.R | 70 | 109 | 111 | 56 | 0.992(0.044) | 1.034(0.058) |
|  |  |  |  |  | RD value, mean(*s.d.*) | |
| Superior longitudinal fasciculus.R | 51 | 119 | 103 | 234 | 0.532(0.025) | 0.502(0.026) |
| Anterior corona radiata.L | 108 | 164 | 79 | 220 | 0.541(0.052) | 0.507(0.039) |
| Body of corpus callosum | 103 | 135 | 100 | 196 | 0.439(0.052) | 0.398(0.040) |
| Posterior thalamic radiation (include optic radiation).L | 102 | 62 | 109 | 158 | 0.542(0.042) | 0.505(0.034) |
| Posterior corona radiata.R | 64 | 65 | 103 | 151 | 0.586(0.039) | 0.551(0.032) |
| Posterior thalamic radiation (include optic radiation).L | 123 | 65 | 92 | 125 | 0.552(0.058) | 0.512(0.042) |
| Superior longitudinal fasciculus.L | 126 | 73 | 93 | 86 | 0.541(0.043) | 0.504(0.039) |
| Body of corpus callosum | 71 | 99 | 109 | 80 | 0.586(0.043) | 0.544(0.050) |
| Sagittal striatum (include inferior logitudinal fasciculus and Inferior fronto-occipital fasciculus).R | 47 | 89 | 64 | 65 | 0.560(0.034) | 0.521(0.049) |
| Splenium of corpus callosum | 72 | 82 | 106 | 64 | 0.616(0.037) | 0.584(0.035) |
| Parietal arcuate fasciculus.L | 126 | 67 | 105 | 64 | 0.559(0.053) | 0.519(0.042) |
| Anterior corona radiata.L | 108 | 165 | 90 | 60 | 0.548(0.041) | 0.514(0.038) |
| Superior longitudinal fasciculus.L | 78 | 61 | 112 | 57 | 0.560(0.043) | 0.520(0.037) |
| Posterior corona radiata.L | 112 | 95 | 112 | 53 | 0.585(0.045) | 0.551(0.041) |
| Sagittal striatum (include inferior logitudinal fasciculus and inferior fronto-occipital fasciculus).R | 52 | 71 | 68 | 52 | 0.536(0.036) | 0.505(0.040) |
|  |  |  |  |  | MD value, mean(*s.d.*) | |
| Body of corpus callosum | 75 | 127 | 106 | 347 | 0.790(0.023) | 0.761(0.028) |
| Splenium of corpus callosum | 112 | 64 | 102 | 328 | 0.786(0.029) | 0.756(0.024) |
| Body of corpus callosum | 76 | 140 | 100 | 215 | 0.783(0.030) | 0.754(0.028) |
| Splenium of corpus callosum | 108 | 99 | 106 | 157 | 0.806(0.028) | 0.776(0.029) |
| Superior longitudinal fasciculus.R | 60 | 116 | 116 | 154 | 0.696(0.026) | 0.670(0.023) |
| Superior longitudinal fasciculus.R | 48 | 105 | 101 | 143 | 0.743(0.028) | 0.717(0.021) |
| Posterior corona radiata.L | 116 | 81 | 114 | 136 | 0.784(0.037) | 0.754(0.029) |
| Superior longitudinal fasciculus.R | 53 | 83 | 105 | 121 | 0.751(0.031) | 0.720(0.026) |
| Body of corpus callosum | 105 | 144 | 99 | 73 | 0.729(0.035) | 0.696(0.034) |
| Frontal arcuate fasciculus. R | 73 | 106 | 128 | 69 | 0.734(0.025) | 0.713(0.024) |
| Superior longitudinal fasciculus.L | 55 | 130 | 93 | 65 | 0.718(0.028) | 0.692(0.026) |
| Parietal arcuate fasciculus.L | 124 | 58 | 102 | 64 | 0.787(0.032) | 0.758(0.025) |
| Posterior thalamic radiata.R | 76 | 62 | 114 | 62 | 0.783(0.030) | 0.754(0.028) |
| Superior longitudinal fasciculus.L | 129 | 81 | 90 | 61 | 0.748(0.032) | 0.720(0.029) |
| Body of corpus callosum | 103 | 135 | 100 | 57 | 0.782(0.041) | 0.744(0.029) |
| Parietal arcuate fasciculus.L | 124 | 70 | 105 | 54 | 0.766(0.039) | 0.734(0.030) |
| Parietal arcuate fasciculus.R | 53 | 58 | 100 | 54 | 0.768(0.032) | 0.739(0.030) |
| Posterior corona radiata.L | 125 | 70 | 98 | 52 | 0.792(0.041) | 0.759(0.033) |

Abbreviations: FA, fractional anisotropy; RD, radial diffusivity; AD, axial diffusivity; MD, mean diffusivity; *s.d.*, standard deviation; L, left; R, right.
