# Supplementary figures and images for: Increased Peripheral Interleukin 10 Relate to White Matter Integrity in Schizophrenia
Source: Front Neurosci. 2019 Feb 7;13:52. doi: 10.3389/fnins.2019.00052 (PMC6374337; doi:10.3389/fnins.2019.00052)

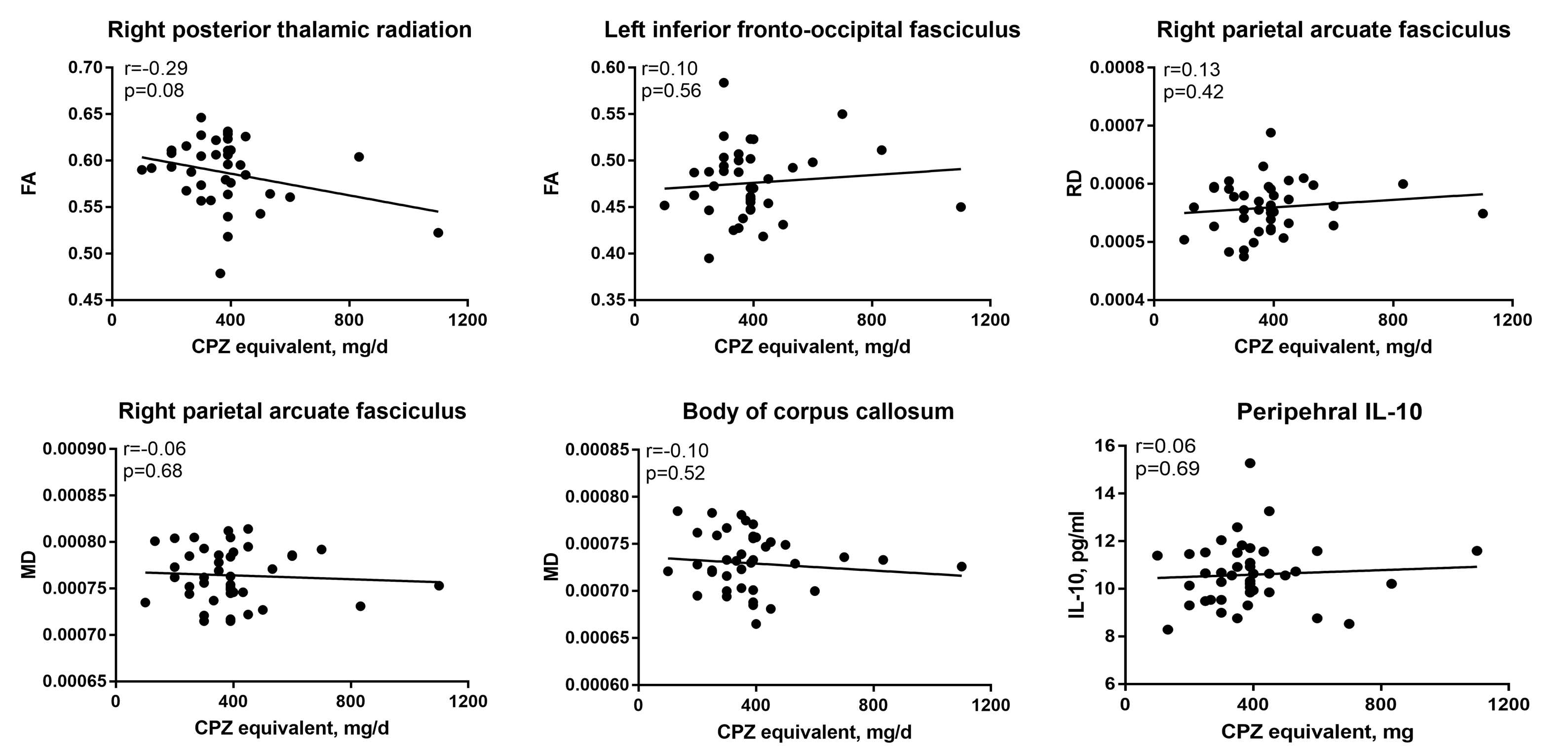

Supplement: FIGURE S1 — Relationship between antipsychotic dosage (CPZ equivalent) and DTI values/ peripheral IL-10 levels. FA, fractional anisotropy; RD, radial diffusivity; MD, mean diffusivity; CPZ, chlorpromazine. [file Image_1.tif]

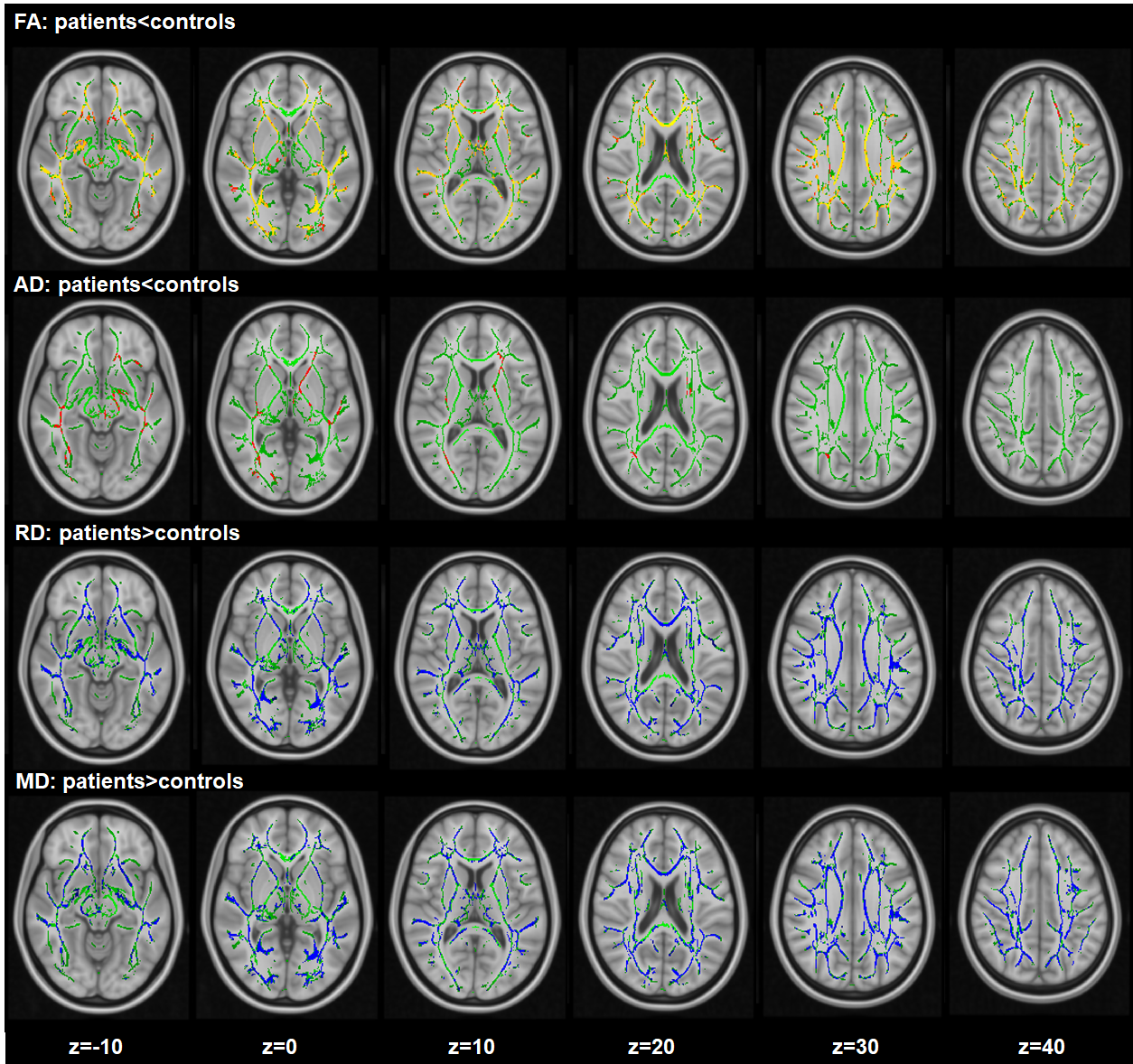

Supplement: FIGURE S2 — Widespread changes of DTI measures in schizophrenia patients compared with healthy controls (peripheral IL-10 as the covariate). Regions with significant decrease are highlighted in yellow-red/orange. Regions with significant increase are highlighted in blue. Results are shown overlaid on the Montreal Neurologic Institute (MNI) template (1 mm). FA, fractional anisotropy; RD, radial diffusivity; AD, axial diffusivity; MD, mean diffusivity. [file Image_2.tif]
