# Supplementary material for: Increased Peripheral Interleukin 10 Relate to White Matter Integrity in Schizophrenia
Source: Front Neurosci. 2019 Feb 7;13:52. doi: 10.3389/fnins.2019.00052 (PMC6374337; doi:10.3389/fnins.2019.00052)
Supplement: Supplementary file 4 [file Data_Sheet_1.PDF]

Supplementary Table 2. Relationship between DTI values and peripheral cytokines.

Regions	IFNγ		IL-1β		IL-2		IL-6		IL-8		TGFβ	
	Patients	Controls	Patients	Controls	Patients	Controls	Patients	Controls	Patients	Controls	Patients	Controls
	β(p*)	β(p*)	β(p*)	β(p*)	β(p*)	β(p*)	β(p*)	β(p*)	β(p*)	β(p*)	β(p*)	β(p*)
**FA**												
Anterior corona radiata.L	-0.005(0.84)	-0.005(0.80)	<-0.001(0.22)	<-0.001(0.30)	<-0.001(0.15)	<0.001(0.23)	0.005(0.03)	<0.001(0.47)	0.003(0.54)	0.003(0.19)	-0.007(0.68)	-0.007(0.89)
Sagittal striatum (include inferior logitudinal fasciculus and inferior fronto-occipital fasciculus).R	-0.005(0.23)	<-0.001(0.23)	<-0.001(0.15)	<-0.001(0.23)	<-0.001(0.32)	<0.001(0.17)	0.004(0.01)	<0.001(0.80)	-0.005(0.98)	0.001(0.62)	-0.007(0.62)	-0.006(0.37)
Body of corpus callosum	-0.005(0.53)	<0.001(0.24)	<-0.001(0.39)	<0.001(0.15)	<-0.001(0.31)	<0.001(0.01)	0.006(0.05)	<0.001(0.12)	-0.004(0.43)	0.004(0.17)	-0.006(0.38)	-0.006(0.30)
Posterior corona radiata.R	-0.005(0.38)	-0.005(0.68)	<-0.001(0.42)	<0.001(0.62)	<-0.001(0.08)	<0.001(0.40)	0.002(0.35)	<0.001(0.51)	<0.001(0.98)	-0.001(0.57)	-0.008(0.96)	-0.006(0.39)
Splenium of corpus callosum	-0.005(0.98)	-0.006(0.97)	<0.001(0.53)	-0.005(0.82)	<-0.001(0.46)	<0.001(0.13)	0.002(0.35)	<-0.001(0.73)	0.001(0.74)	0.001(0.55)	-0.006(0.38)	-0.006(0.66)
Sagittal striatum (include inferior logitudinal fasciculus and inferior fronto-occipital fasciculus).L	-0.005(0.43)	<-0.001(0.58)	<-0.001(0.37)	<-0.001(0.60)	<-0.001(0.74)	<0.001(0.19)	<0.002(0.50)	<-0.001(0.73)	-0.002(0.57)	0.001(0.54)	-0.008(0.98)	-0.008(0.99)
Splenium of corpus callosum	-0.005(0.73)	<0.001(0.14)	<-0.001(0.32)	<0.001(0.30)	<-0.001(0.11)	<0.001(0.09)	<0.001(0.96)	<0.001(0.58)	-0.002(0.47)	0.002(0.57)	-0.007(0.79)	-0.007(0.92)
Body of corpus callosum	-0.005(0.77)	-0.005(0.95)	<-0.001(0.38)	-0.006(0.99)	<-0.001(0.67)	<0.001(0.05)	<-0.001(0.80)	<-0.001(0.94)	-0.005(0.38)	0.002(0.35)	-0.007(0.71)	-0.006(0.51)
Temporal arcuate fasciculus.L	-0.005(0.40)	<0.001(0.02)	<-0.001(0.34)	<0.001(0.29)	<0.001(0.42)	0.001(0.02)	0.003(0.23)	0.002(0.35)	<0.001(0.91)	0.005(0.16)	-0.007(0.88)	-0.009(0.64)
Posterior thalamic radiation (include optic radiation).L	-0.005(0.90)	-0.007(0.14)	<0.001(0.82)	-0.007(0.70)	<0.001(0.73)	-0.007(0.49)	0.002(0.44)	-0.006(0.11)	0.002(0.66)	-0.006(0.12)	-0.007(0.73)	-0.009(0.54)
Inferior fronto-occipital fasciculus.L	-0.005(0.21)	<-0.001(0.67)	<-0.001(0.46)	-0.005(0.97)	<0.001(0.35)	<0.001(0.46)	<-0.001(0.01)	-0.002(0.14)	-0.007(0.07)	-0.002(0.53)	-0.006(0.40)	-0.006(0.46)
Posterior thalamic radiation (include optic radiation).R	-0.005(0.86)	<-0.001(0.52)	<0.001(0.11)	<0.001(0.40)	<0.001(0.57)	<0.001(0.07)	<0.001(0.77)	-0.005(0.99)	<0.001 (0.88)	0.001(0.81)	-0.006(0.15)	-0.007(0.95)
Superior longitudinal fasciculus.R	-0.005(0.40)	-0.005(0.77)	<0.001(0.49)	-0.005(0.96)	-0.005(0.96)	<0.001(0.07)	<-0.001(0.65)	<0.001(0.60)	<0.001(0.92)	0.004(0.19)	-0.007(0.89)	-0.007(0.84)
Splenium of corpus callosum	-0.005(0.87)	<-0.001(0.38)	<0.001(0.55)	<-0.001(0.14)	<0.001(0.53)	<0.001(0.40)	<-0.001(0.90)	-0.004(0.01)	0.004(0.48)	0.002(0.51)	-0.006(0.07)	-0.006(0.73)
Body of corpus callosum	-0.005(0.76)	<0.001(0.11)	-0.001(0.04)	<0.001(0.17)	<-0.001(0.16)	<0.001(0.11)	0.004(0.18)	<0.001(0.72)	0.003(0.46)	0.001(0.67)	-0.006(0.18)	-0.001(0.80)
**AD**												
Superior corona radiata.R	-0.007(0.08)	-0.007(0.92)	-0.007(0.78)	-0.007(0.70)	-0.007(0.66)	-0.007(0.58)	-0.005(0.01)	-0.006(0.73)	-0.005(0.11)	-0.007(0.95)	-0.009(0.01)	-0.009(0.63)
Anterior corona radiata.R	-0.008(0.38)	-0.007(0.41)	-0.007(0.75)	-0.007(0.18)	-0.007(0.25)	-0.007(0.57)	-0.006(0.15)	-0.007(0.84)	-0.006(0.74)	-0.006(0.47)	-0.009(0.59)	-0.009(0.83)
Superior corona radiata.R	-0.007(0.15)	-0.008(0.93)	-0.007(0.81)	-0.007(0.51)	-0.007(0.90)	-0.007(0.14)	-0.006(0.61)	-0.006(0.60)	-0.006(0.39)	-0.006(0.11)	-0.01(0.91)	-0.009(0.11)
Superior corona radiata.R	-0.008(0.56)	-0.007(0.16)	-0.008(0.66)	-0.007(0.79)	-0.007(0.63)	-0.007(0.23)	-0.008(0.99)	-0.006(0.48)	-0.006(0.70)	-0.006(0.36)	-0.009(0.32)	-0.009(0.45)

**RD**												
Superior corona radiata.R	-0.008(0.23)	-0.007(0.31)	-0.007(0.46)	-0.007(0.28)	-0.007(0.53)	-0.007(0.10)	-0.006(0.93)	-0.006(0.96)	-0.006(0.33)	-0.006(0.25)	-0.009(0.94)	-0.01(0.96)
Superior longitudinal fasciculus.R	-0.008(0.66)	-0.007(0.27)	-0.007(0.23)	-0.007(0.16)	-0.006(0.14)	-0.007(0.30)	-0.006(0.01)	-0.006(0.93)	-0.006(0.33)	-0.006(0.23)	-0.009(0.38)	-0.009(0.63)
Anterior corona radiata.L	-0.008(0.38)	-0.007(0.30)	-0.007(0.18)	-0.007(0.19)	-0.006(0.15)	-0.006(0.01)	-0.006(0.02)	-0.006(0.28)	-0.006(0.83)	-0.006(0.13)	-0.009(0.16)	-0.009(0.40)
Body of corpus callosum	-0.008(0.36)	-0.007(0.09)	-0.007(0.74)	-0.007(0.47)	-0.008(0.95)	-0.007(0.03)	-0.006(0.38)	-0.008(0.96)	-0.006(0.38)	-0.006(0.06)	-0.009(0.53)	-0.009(0.67)
Posterior thalamic radiation (include optic radiation).L	-0.008(0.68)	-0.007(0.22)	-0.007(0.11)	-0.007(0.84)	-0.006(0.01)	-0.007(0.79)	-0.006(0.11)	-0.006(0.42)	-0.007(0.90)	-0.006(0.25)	-0.01(0.83)	-0.009(0.16)
Posterior corona radiata.R	-0.007(0.66)	-0.006(0.55)	-0.006(0.32)	-0.007(0.60)	-0.006(0.55)	-0.007(0.37)	-0.005(0.35)	-0.006(0.80)	-0.005(0.16)	-0.005(0.61)	-0.009(0.51)	-0.008(0.34)
Posterior thalamic radiation (include optic radiation).L	-0.008(0.77)	-0.007(0.31)	-0.007(0.84)	-0.007(0.36)	-0.007(0.92)	-0.006(0.02)	-0.006(0.24)	-0.007(0.72)	-0.006(0.72)	-0.006(0.37)	-0.01(0.90)	-0.009(0.39)
Superior longitudinal fasciculus.L	-0.008(0.71)	-0.007(0.62)	-0.006(0.03)	-0.007(0.49)	-0.006(0.06)	-0.007(0.56)	-0.006(0.04)	-0.006(0.25)	-0.006(0.26)	-0.006(0.66)	-0.009(0.23)	-0.01(0.89)
Body of corpus callosum	-0.007(0.11)	-0.007(0.15)	-0.007(0.85)	-0.007(0.69)	-0.007(0.53)	-0.006(0.06)	-0.006(0.52)	-0.006(0.37)	-0.007(0.96)	-0.006(0.16)	-0.009(0.20)	-0.01(0.88)
Sagittal striatum (include inferior logitudinal fasciculus and Inferior fronto-occipital fasciculus).R	-0.007(0.07)	-0.007(0.33)	-0.007(0.65)	-0.007(0.41)	-0.008(0.98)	-0.007(0.21)	-0.006(0.08)	-0.006(0.43)	-0.007(0.85)	-0.006(0.26)	-0.009(0.15)	-0.009(0.31)
Splenium of corpus callosum	-0.008(0.83)	-0.007(0.65)	-0.007(0.36)	-0.007(0.71)	-0.006(0.04)	-0.007(0.55)	-0.006(0.44)	-0.006(0.61)	-0.006(0.65)	-0.006(0.56)	-0.01(0.53)	-0.01(0.81)
Parietal arcuate fasciculus.L	-0.007(0.08)	-0.008(0.88)	-0.007(0.28)	-0.007(0.69)	-0.006(0.16)	-0.007(0.59)	-0.007(0.92)	-0.006(0.56)	-0.007(0.90)	-0.006(0.77)	-0.01(0.86)	-0.009(0.14)
Anterior corona radiata.L	-0.008(0.84)	-0.007(0.12)	-0.007(0.33)	-0.007(0.74)	-0.006(0.27)	-0.007(0.51)	-0.006(0.006)	-0.006(0.43)	-0.006(0.35)	-0.006(0.23)	-0.009(0.19)	-0.009(0.84)
Superior longitudinal fasciculus.L	-0.008(0.35)	-0.007(0.21)	-0.007(0.79)	-0.007(0.75)	-0.007(0.44)	-0.007(0.23)	-0.006(0.18)	-0.006(0.37)	-0.006(0.21)	-0.006(0.16)	-0.009(0.55)	-0.009(0.12)
Posterior corona radiata.L	-0.008(0.30)	-0.007(0.51)	-0.007(0.28)	-0.007(0.56)	-0.006(0.12)	-0.007(0.50)	-0.006(0.03)	-0.007(0.61)	-0.006(0.65)	-0.007(0.92)	-0.01(0.81)	-0.009(0.35)
Sagittal striatum (include inferior logitudinal fasciculus and inferior fronto-occipital fasciculus).R	-0.008(0.34)	-0.007(0.64)	-0.007(0.17)	-0.007(0.46)	-0.006(0.16)	-0.007(0.16)	-0.006(0.11)	-0.008(0.97)	-0.006(0.68)	-0.006(0.70)	-0.01(0.56)	-0.009(0.45)
**MD**												
Body of corpus callosum	-0.008(0.64)	-0.009(0.98)	-0.007(0.63)	-0.007(0.46)	-0.007(0.32)	-0.007(0.13)	-0.006(0.47)	-0.007(0.50)	-0.006(0.60)	-0.006(0.20)	-0.009(0.20)	-0.009(0.19)
Splenium of corpus callosum	-0.008(0.54)	-0.008(0.82)	-0.007(0.78)	-0.007(0.22)	-0.007(0.51)	-0.007(0.61)	-0.006(0.23)	-0.007(0.45)	-0.006(0.71)	-0.006(0.63)	-0.01(0.81)	-0.009(0.09)
Body of corpus callosum	-0.008(0.22)	-0.008(0.94)	-0.007(0.57)	-0.008(0.87)	-0.006(0.13)	-0.007(0.46)	-0.007(0.57)	-0.007(0.84)	-0.006(0.16)	-0.006(0.90)	-0.01(0.75)	-0.009(0.76)
Splenium of corpus callosum	-0.008(0.83)	-0.008(0.75)	-0.007(0.43)	-0.007(0.77)	-0.007(0.23)	-0.007(0.13)	-0.006(0.04)	-0.007(0.77)	-0.006(0.44)	-0.006(0.44)	-0.01(0.41)	-0.009(0.02)
Superior longitudinal fasciculus.R	-0.008(0.17)	-0.008(0.88)	-0.007(0.31)	-0.007(0.33)	-0.007(0.34)	-0.007(0.69)	-0.007(0.65)	-0.007(0.76)	-0.007(0.86)	-0.006(0.45)	-0.01(0.89)	-0.009(0.39)
Superior longitudinal fasciculus.R	-0.009(0.56)	-0.008(0.68)	-0.007(0.68)	-0.007(0.22)	-0.006(0.15)	-0.007(0.26)	-0.006(0.57)	-0.007(0.77)	-0.006(0.74)	-0.008(0.96)	-0.009(0.28)	-0.009(0.25)
Posterior corona radiata.L	-0.009(0.93)	-0.008(0.81)	-0.007(0.10)	-0.008(0.91)	-0.006(0.06)	-0.007(0.14)	-0.007(0.03)	-0.007(0.57)	-0.006(0.08)	-0.006(0.21)	-0.01(0.93)	-0.009(0.10)
Superior longitudinal fasciculus.R	-0.008(0.93)	-0.008(0.62)	-0.007(0.72)	-0.007(0.48)	-0.006(0.09)	-0.007(0.72)	-0.006(0.04)	-0.007(0.74)	-0.006(0.22)	-0.007(0.96)	-0.01(0.61)	-0.009(0.33)
Body of corpus callosum	-0.008(0.67)	-0.007(0.89)	-0.007(0.70)	-0.007(0.23)	-0.006(0.10)	-0.008(0.91)	-0.006(0.003)	-0.007(0.71)	-0.006(0.04)	-0.007(0.94)	-0.01(0.04)	-0.009(0.33)

Frontal arcuate fasciculus. R	-0.008(0.77)	-0.008(0.93)	-0.008(0.92)	-0.007(0.18)	-0.007(0.88)	-0.007(0.52)	-0.006(0.48)	-0.007(0.57)	-0.006(0.26)	-0.007(0.63)	-0.01(0.42)	-0.009(0.48)
Superior longitudinal fasciculus.L	-0.008(0.80)	-0.007(0.56)	-0.007(0.15)	-0.008(0.54)	-0.006(0.20)	-0.007(0.52)	-0.006(0.14)	-0.007(0.57)	-0.007(0.91)	-0.006(0.35)	-0.01(0.88)	-0.009(0.58)
Parietal arcuate fasciculus.L	-0.009(0.88)	-0.007(0.31)	-0.007(0.41)	-0.008(0.70)	-0.007(0.29)	-0.007(0.65)	-0.007(0.06)	-0.007(0.16)	-0.006(0.29)	-0.006(0.84)	-0.01(0.92)	-0.009(0.11)
Posterior thalamic radiata.R	-0.008(0.22)	-0.008(0.94)	-0.007(0.57)	-0.007(0.87)	-0.006(0.13)	-0.007(0.46)	-0.006(0.57)	-0.007(0.45)	-0.006(0.16)	-0.007(0.90)	-0.01(0.75)	-0.01(0.76)
Superior longitudinal fasciculus.L	-0.007(0.69)	-0.007(0.52)	-0.007(0.20)	-0.007(0.66)	-0.006(0.11)	-0.008(0.84)	-0.007(0.07)	-0.007(0.57)	-0.006(0.68)	-0.006(0.63)	-0.01(0.61)	-0.009(0.11)
Body of corpus callosum	-0.008(0.34)	-0.008(0.74)	-0.007(0.13)	-0.007(0.69)	-0.006(0.03)	-0.007(0.14)	-0.005(0.002)	-0.007(0.74)	-0.006(0.14)	-0.006(0.33)	-0.009(0.06)	-0.009(0.51)
Parietal arcuate fasciculus.L	-0.005(0.22)	-0.007(0.55)	-0.007(0.44)	-0.007(0.30)	-0.006(0.33)	-0.007(0.67)	-0.006(0.42)	-0.007(0.93)	-0.006(0.45)	-0.006(0.57)	-0.01(0.98)	-0.009(0.32)
Parietal arcuate fasciculus.R	-0.008(0.46)	-0.007(0.11)	-0.008(0.91)	-0.008(0.88)	-0.007(0.31)	-0.007(0.62)	-0.007(0.08)	-0.007(0.82)	-0.006(0.63)	-0.006(0.47)	-0.01(0.59)	-0.009(0.05)
Posterior corona radiata.L	-0.008(0.40)	-0.007(0.27)	-0.007(0.11)	-0.007(0.47)	-0.006(0.23)	-0.007(0.58)	-0.007(0.24)	-0.007(0.90)	-0.007(0.84)	-0.006(0.43)	-0.009(0.43)	-0.01(0.81)

Abbreviations: IFNγ: interferon γ; TGFβ: tumor growth factor β; FA: fractional anisotropy; RD: radial diffusivity; AD: axial diffusivity; MD: mean diffusivity; L: left; R: right. p*: uncorrected p value.
